# Sexual identity, attraction and behaviour in Britain: The implications of using different dimensions of sexual orientation to estimate the size of sexual minority populations and inform public health interventions

**DOI:** 10.1371/journal.pone.0189607

**Published:** 2018-01-02

**Authors:** Rebecca S. Geary, Clare Tanton, Bob Erens, Soazig Clifton, Philip Prah, Kaye Wellings, Kirstin R. Mitchell, Jessica Datta, Kirsten Gravningen, Elizabeth Fuller, Anne M. Johnson, Pam Sonnenberg, Catherine H. Mercer

**Affiliations:** 1 Institute for Global Health, University College London, London, United Kingdom; 2 Department of Health Services Research & Policy, London School of Hygiene & Tropical Medicine, London, United Kingdom; 3 Department of Social and Environmental Health Research, London School of Hygiene & Tropical Medicine, London, United Kingdom; 4 MRC/CSO Social and Public Health Sciences Unit, Institute of Health and Wellbeing, University of Glasgow, Glasgow, United Kingdom; 5 Department of Microbiology & Infection Control, University Hospital of North Norway, Tromsø, Norway; 6 NatCen Social Research, London, United Kingdom; University of Westminster, UNITED KINGDOM

## Abstract

**Background:**

Sexual orientation encompasses three dimensions: sexual identity, attraction and behaviour. There is increasing demand for data on sexual orientation to meet equality legislation, monitor potential inequalities and address public health needs. We present estimates of all three dimensions and their overlap in British men and women, and consider the implications for health services, research and the development and evaluation of public health interventions.

**Methods:**

Analyses of data from Britain’s third National Survey of Sexual Attitudes and Lifestyles, a probability sample survey (15,162 people aged 16–74 years) undertaken in 2010–2012.

**Findings:**

A lesbian, gay or bisexual (LGB) identity was reported by 2·5% of men and 2·4% of women, whilst 6·5% of men and 11·5% of women reported any same-sex attraction and 5·5% of men and 6·1% of women reported ever experience of same-sex sex. This equates to approximately 547,000 men and 546,000 women aged 16–74 in Britain self-identifying as LGB and 1,204,000 men and 1,389,000 women ever having experience of same-sex sex. Of those reporting same-sex sex in the past 5 years, 28% of men and 45% of women identified as heterosexual.

**Interpretation:**

There is large variation in the size of sexual minority populations depending on the dimension applied, with implications for the design of epidemiological studies, targeting and monitoring of public health interventions and estimating population-based denominators. There is also substantial diversity on an individual level between identity, behaviour and attraction, adding to the complexity of delivering appropriate services and interventions.

## Introduction

Sexual orientation can be seen as encompassing three dimensions: sexual identity, attraction and behaviour[1]. In the UK, there is an increasing demand for data on sexual orientation to meet equality legislation[2]; and data on health outcomes by sexual orientation, are necessary to understand the health and wellbeing needs of different population groups, and to monitor potential inequalities.

Disparities in health, particularly sexual and mental health, have been reported between those identifying as lesbian, gay and bisexual (LGB) and those identifying as heterosexual[3–8]. The UK Office for National Statistics (ONS) introduced a question on sexual identity in their social surveys[1] in 2009 to provide benchmark population estimates for the LGB populations, and to further the aim of monitoring equality of opportunity by sexual orientation in areas such as education, employment and the provision of services. However, including a suite of questions to capture the three dimensions of sexual orientation was deemed too costly and too burdensome for participants[1]. Sexual identity was considered the dimension of sexual orientation most relevant to measure given its relation to experiences of disadvantage and discrimination[1]. However, the implication of restricting benchmark population estimates to one particular dimension of sexual orientation has not been assessed. Britain’s third National Survey of Sexual Attitudes and Lifestyles, Natsal-3, collected data on sexual identity, attraction and behaviour from a representative sample of the population[9], making it uniquely placed to quantify the impact of applying different definitions of sexual orientation at a population level. This paper quantifies these three dimensions by age and sex, examines the extent to, and the way in which they overlap, and considers the implications of using different dimensions for estimating the size of sexual minority populations in Britain for health services, research and the development and evaluation of public health interventions.

## Materials and methods

### Participants and procedures

Full details of the methods used in Natsal-3 have been reported elsewhere[9,10]. Briefly, we used a multistage, clustered, stratified probability sample design. A total of 15,162 men and women aged 16–74 years, resident in a private household in Britain were interviewed between September 2010 and August 2012. The response rate was 57·7%.

Participants were interviewed through a combination of face-to-face computer-assisted personal interview (CAPI) and computer-assisted self-interview (CASI) for the more sensitive questions. Questions pertinent to this paper are detailed in Box 1. The Natsal-3 interview began with CAPI questions in which all participants, regardless of their sexual experience to date, were shown two cards sequentially, with questions about sexual attraction and sexual experience (Box 1, Initial CAPI questions). Participants were asked to read these cards to themselves, and tell the interviewer the letter that best represented their answer.

**Box 1. Sexual orientation questions**

**Initial CAPI questions for *men* (*women*)^a^**

***Sexual attraction^b^***

I have felt **sexually attracted**…

(K) Only to *females (males*), never to *males (females*)
(C) More often to *females (males*), and at least once to a *male (female*)
(F) About equally often to *females (males*) and to *males (females*)
(L) More often to *males (females*), and at least once to a *female (male*)
(D) Only ever to *males (females*), never to *females (males*)
(N) I have never felt sexually attracted to anyone at all

***Sexual experience scale***

**Sexual experience** is any kind of contact with another person that you felt was

sexual (it could be just kissing or touching, or intercourse or any other form of sex).

I have had some **sexual experience**…

(R) Only with *females (males*) (or a *female (male*)), never with a *male (female*)
(Q) More often with *females (males*), and at least once with a *male (female*)
(T) About equally often with *females (males*) and with *males (females*)
(B) More often with *males (females*), and at least once with a *female (male*)
(Z) Only with *males (females*) (or a *male (female*)), never with a *female (male*)
(W) I have never had any sexual experience with anyone at all

**CASI questions for *men (women)***

***Same-sex experience ever***

Have you ever had any kind of sexual experience or sexual contact with a *man (woman*)?

Please say 'yes' here, even if it was a long time ago or did not involve contact

with the *genital area/penis (genital area/vagina*).

1    Yes
2    No

***Same-sex sex ever***

[Those answering ‘yes’ were then asked:]

Have you had sex with a *man (woman*) involving *genital area/penis*

*(genital area/vaginal*) contact?

(That is *oral or anal (oral)* sex or any other contact involving the genital area.)

1    Yes
2    No

**Final CAPI questions**

***Sexual identity***

Which of the options on this card best describes how you think of yourself?

(R) Heterosexual / Straight
(H) Gay/ Lesbian
(I) Bisexual
(J) Other

^a^ *Italics* indicate where text differed for men and women with text specific to men appearing in italics and alternative text for women as italics in brackets.

^b^ ***Bold italics*** indicate how these questions were refferred to in this article.

Participants who reported having any sexual experience, or who refused to answer this question, were invited to complete the CASI, which included questions on same-sex sexual experience (Box 1, CASI questions). In addition, the CASI asked questions about the number of same-sex partners, including none, in different time periods (ever and past 5 years)[11]. After the CASI, all participants completed another CAPI section, which asked demographic questions, including the 2009 harmonised ONS question on sexual identity[1], (again shown to participants on a card) (Box 1, Final CAPI questions).

### Statistical analysis

We conducted analyses using the complex survey functions of Stata (version 14) to incorporate weighting, clustering, and stratification of the Natsal-3 data. We present descriptive statistics by sex and age-group to examine variation in reporting of different dimensions of sexual orientation, and describe the extent to which they overlap. We apply our estimates to ONS 2011 census population estimates (which coincide with midway through Natsal-3 fieldwork) of men and women aged 16–74 years in Britain to quantify the size of sexual minority populations according to the different dimensions of sexual orientation.

### Ethics statement

All Natsal-3 participants were given an information leaflet to read prior to participation. In line with standard practice for UK surveys, and in response to evidence suggesting that signing a consent form might lead to a greater sense of obligation to complete the interview, we obtained verbal rather than written consent [12]. We ensured procedures for obtaining verbal informed consent via our interviewer training and protocols: interviewers were trained to make sure that participants had read the information leaflet and had the opportunity to discuss the study fully before the interview began; and at the beginning of each interview, interviewers were prompted (on screen) to remind participants that they could choose not to answer any question. Interviewers had to confirm in the computer programme that respondents had read the information leaflet before commencing the interview. The Natsal-3 study, was approved by the Oxfordshire Research Ethics Committee A (reference: 09/H0604/27). All participants provided their own consent to participate, however for 16–17 year olds living at home, a parent/guardian provided additional verbal assent for participation.

## Results

### Dimensions of sexual orientation

Overall, 2·5% of 16–74 year old men and 2·4% of women self-identified as lesbian, gay or bisexual; 6·5% of men and 11·5% women reported any same-sex sexual attraction; 5·5% of men and 6·1% of women reported same-sex sex ever, and a further 2·4% of men and 5·3% of women reported same-sex experience but never with genital contact (7·9% and 11·4% in total; Table 1).

**Table 1:** Sexual identity, attraction and behaviours by age group among men and women aged 16–74 years.

	16–24		25–34		35–44		45–54		55–64		65–74		p-value (trend)	Total	
**Men**															
*Denominators (unweighted*, *weighted)*	*1729*	*1238*	*1525*	*1374*	*806*	*1425*	*794*	*1413*	*772*	*1197*	*667*	*860*	* *	*6293*	*7508*
**Sexual Identity**^**a**^													0.012		
Heterosexual/Straight	96.7%	[95.6–97.6]	96.5%	[95.3–97.3]	97.7%	[95.9–98.7]	96.7%	[95.1–97.7]	96.8%	[95.4–97.8]	99.0%	[97.8–99.6]		97.1%	[96.6–97.6]
Gay/Lesbian	1.5%	[1.0–2.3]	2.4%	[1.7–3.4]	1.3%	[0.7–2.6]	1.8%	[1.1–2.8]	1.3%	[0.7–2.1]	0.2%	[0.0–0.8]		1.5%	[1.2–1.9]
Bisexual	1.5%	[1.0–2.3]	0.7%	[0.4–1.2]	0.9%	[0.3–2.8]	1.2%	[0.7–2.3]	1.3%	[0.7–2.4]	0.5%	[0.2–1.4]		1.0%	[0.8–1.4]
Other	0.3%	[0.1–0.7]	0.5%	[0.2–1.0]	0.1%	[0.0–0.4]	0.3%	[0.1–1.8]	0.6%	[0.3–1.6]	0.3%	[0.0–2.0]		0.3%	[0.2–0.6]
**Any same-sex attraction**	7.0%	[5.8–8.5]	7.9%	[6.5–9.4]	4.7%	[3.4–6.5]	6.4%	[4.9–8.4]	8.2%	[6.4–10.5]	4.2%	[3.0–6.0]	0.187	6.5%	[5.8–7.2]
**Same-sex experience but never with genital contact**^**b**^	3.0%	[2.2–4.1]	1.9%	[1.3–2.9]	2.6%	[1.5–4.5]	2.0%	[1.2–3.4]	2.5%	[1.6–4.0]	2.1%	[1.2–3.7]	0.508	2.4%	[1.9–2.9]
**Same-sex sex ever**^**b**^^,^^**c**^	4.0%	[3.1–5.2]	5.7%	[4.6–7.1]	4.7%	[3.3–6.6]	7.2%	[5.5–9.3]	7.3%	[5.6–9.5]	3.4%	[2.3–5.1]	0.131	5.5%	[4.9–6.2]
**Opposite-sex sex ever**^**d**^	86.3%	[84.3–88.0]	95.6%	[94.1–96.7]	98.9%	[97.7–99.5]	98.5%	[97.4–99.1]	98.5%	[97.5–99.1]	98.7%	[97.4–99.3]	<0.001	96.1%	[95.5–96.5]
**Same- and opposite-sex sex**^**e**^	2.9%	[2.2–3.9]	4.2%	[3.2–5.4]	3.9%	[2.7–5.6]	6.4%	[4.8–8.4]	6.9%	[5.3–9.1]	3.2%	[2.1–4.9]	0.002	4.7%	[4.1–5.3]
**Same-sex sex in the past 5 years**^**d**^	2.9%	[2.1–3.9]	3.5%	[2.6–4.7]	2.3%	[1.4–3.7]	2.9%	[1.9–4.3]	2.3%	[1.3–3.8]	0.9%	[0.4–2.0]	0.003	2.6%	[2.1–3.0]
	16–24		25–34		35–44		45–54		55–64		65–74		p-value (trend)	Total	
**Women**															
*Denominators (unweighted*, *weighted)*	*2140*	*1207*	*2487*	*1380*	*1215*	*1455*	*1123*	*1443*	*1030*	*1235*	*874*	*935*	* *	*8869*	*7654*
**Sexual Identity**													<0.001		
Heterosexual/Straight	95.0%	[93.7–96.0]	96.6%	[95.8–97.2]	96.8%	[95.6–97.7]	97.4%	[96.3–98.2]	99.1%	[98.2–99.5]	99.4%	[98.6–99.7]		97.3%	[96.9–97.6]
Gay/Lesbian	0.9%	[0.5–1.4]	1.2%	[0.8–1.8]	1.5%	[0.9–2.4]	1.1%	[0.7–1.9]	0.8%	[0.4–1.7]	0.1%	[0.0–0.6]		1.0%	[0.8–1.2]
Bisexual	3.5%	[2.7–4.5]	2.0%	[1.5–2.7]	1.4%	[0.8–2.2]	1.0%	[0.5–1.9]	0.0%	0.00%	0.3%	[0.1–1.0]		1.4%	[1.2–1.7]
Other	0.7%	[0.3–1.4]	0.2%	[0.1–0.4]	0.3%	[0.1–0.8]	0.5%	[0.2–1.2]	0.1%	[0.0–0.5]	0.3%	[0.1–1.1]		0.3%	[0.2–0.5]
**Any same-sex attraction**	17.2%	[15.4–19.1]	17.2%	[15.6–18.9]	13.6%	[11.7–15.8]	9.6%	[7.9–11.6]	5.3%	[4.0–7.0]	3.4%	[2.3–5.0]	<0.001	11.5%	[10.7–12.2]
**Same-sex experience but never with genital contact**^**b**^	11.3%	[9.8–13.0]	9.3%	[7.9–10.8]	4.0%	[3.0–5.4]	2.2%	[1.5–3.2]	3.0%	[2.1–4.3]	1.4%	[0.8–2.6]	<0.001	5.3%	[4.8–5.8]
**Same-sex sex ever**^**b**^^,^^**c**^	7.6%	[6.4–9.0]	8.8%	[7.7–10.1]	7.4%	[6.0–9.2]	6.6%	[5.3–8.3]	3.5%	[2.4–5.0]	0.8%	[0.4–1.4]	<0.001	6.1%	[5.6–6.7]
**Opposite-sex sex ever**^**d**^	85.4%	[83.5–87.0]	98.2%	[97.5–98.8]	99.8%	[99.4–99.9]	99.5%	[99.0–99.7]	99.2%	[98.6–99.5]	99.3%	[98.7–99.7]	<0.001	97.0%	[96.7–97.4]
**Same- and opposite-sex sex**^**e**^	7.2%	[6.0–8.5]	8.6%	[7.4–9.9]	7.3%	[5.9–9.1]	6.4%	[5.1–8.1]	3.3%	[2.3–4.9]	0.7%	[0.3–1.3]	<0.001	5.9%	[5.4–6.5]
**Same-sex sex in the past 5 years**^**f**^	6.2%	[5.1–7.4]	4.7%	[3.9–5.6]	3.5%	[2.6–4.9]	2.7%	[1.9–3.8]	1.0%	[0.5–2.1]	0.1%	[0.0–0.5]	<0.001	3.2%	[2.8–3.6]

^a^ Estimates for sexual identity among women aged 16–24 years differ slightly from those published in Mercer et al. Lancet 2013;382:1781–94 due to error.

^b^ Those reporting same-sex experience with genital contact (ever) may also have reported same-sex experience without genital contact but are not included in the same-sex experience but never with genital contact group.

^c^ Same-sex experience with genital contact

^d^ Opposite-sex experience with genital contact

^e^ Same- and opposite-sex experience with genital contact

^f^ 1+ same-sex partner in the past 5 years

**Fig 1:**
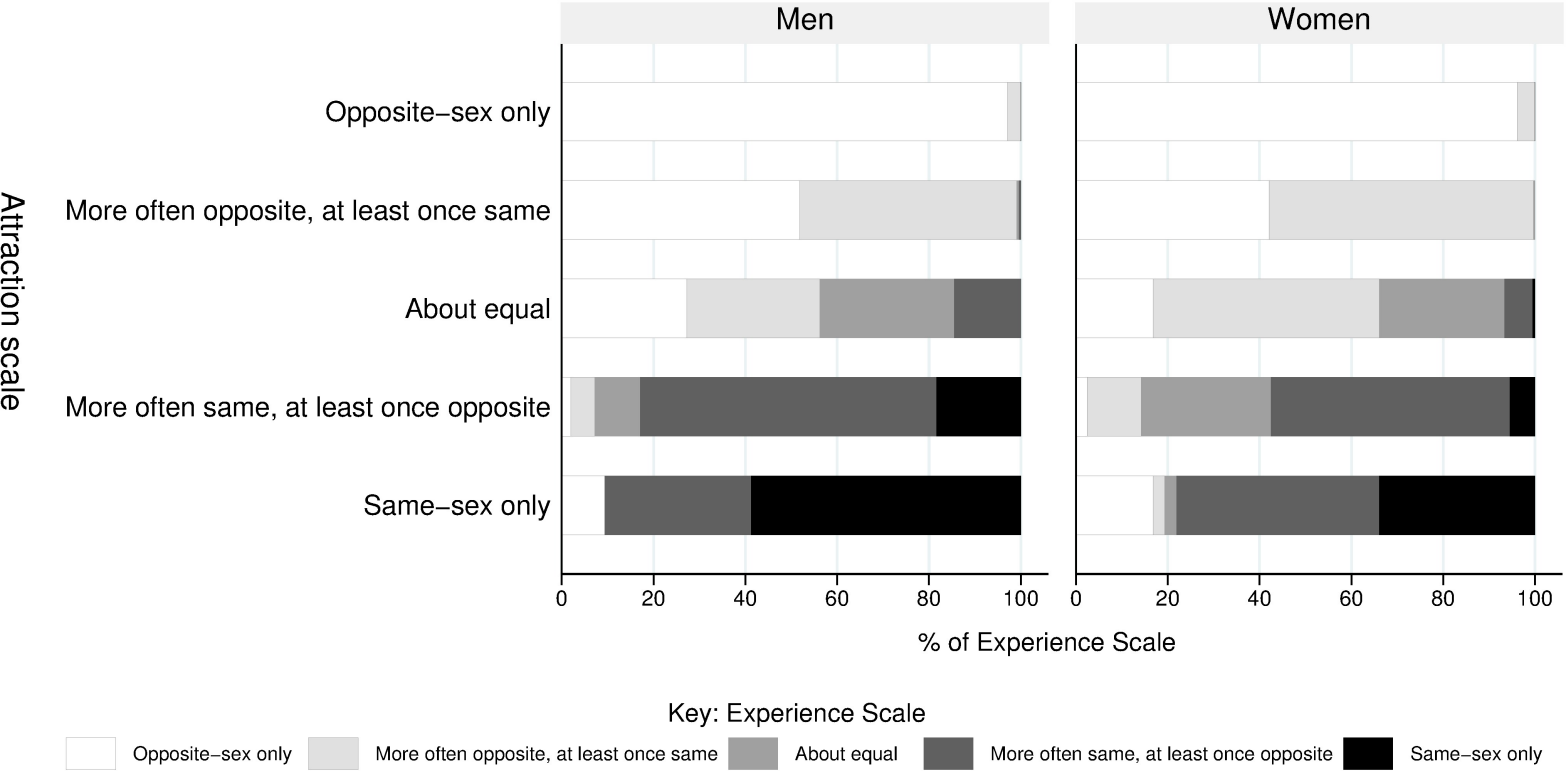
Opposite and same-sex experience by increasing degree of same-sex attraction by sex, among those aged 16–74 years. Men and women who reported either never having felt sexually attracted to anyone or never having had sexual experience with anyone were excluded from the denominator.

After *only* opposite-sex attraction or experience, the most common response to the sexual attraction and experience scale questions was “More often to/with (someone of the opposite sex), and at least once to/with a (same-sex person)”. Men were more likely than women to report *only* same-sex attraction (0·9% vs. 0·4%, S1 Table) and experience (0·7% vs. 0·2%) although prevalence was low. Of those who were more often attracted to people of the opposite sex, but had been attracted to a person of the same sex at least once, women more commonly reported same-sex experience than men (58·0% vs. 48·2%, p = 0·016, Fig 1). However, among those who were more often, or exclusively, attracted to people of the same sex, men were more likely than women to report only same-sex experience (37·8% vs. 14·6%, p = 0·0006, Fig 1).

### Dimensions of sexual orientation by age and sex

With the exception of the oldest age group, there was little difference in reported sexual identity by age in men, while younger women (aged 16–24 years) were more likely to report identifying as bisexual (3·5%) than older women (2·0% among women aged 25–34 years and declining further with age (Table 1)). Similarly, the percentage of women reporting having felt sexually attracted to someone of the same sex was higher among younger women (17·2% to 3·4% among those aged 16–34 and 65–74 years) but there were no such age differences for men. Women aged 16–34 years were more likely than men of the same age to report both “same-sex experience but never with genital contact” (11·3% vs. 3·0% among those aged 16–24 years) and same-sex sex (7·6% vs. 4·0% among 16–24 year olds). Experience of same-sex sex in the last five years was more frequently reported by 16–24 year old women than men in that age group (6·2% vs 2·9%). Among women, reporting of same-sex experience without any genital contact and same-sex sex (ever and in the past 5 years) declined with age. However, reporting of these experiences did not vary by age among men.

### Sexual identity by different measures of sexual attraction & behaviour

Among those reporting any same-sex attraction, men more commonly identified as gay than did women as lesbian (23·0% vs. 8·5%, Table 2). The majority of those reporting same-sex *experience* ever but never with genital contact identified as heterosexual (92·1% of men and 95·2% of women aged 16–74 years). Of those reporting same-sex *sex* ever, 59·5% of men and 65·3% of women identified as heterosexual and men were more likely to identify as gay than women were as lesbian (26·3% vs 15·9% respectively).

**Table 2:** Sexual identity by different measures of sexual attraction and behaviour among men and women aged 16–74 years.

	Men		Women	
*Denominators (all aged 16–74 years)(unweighted*, *weighted)*	*6293*	*7508*	*8869*	*7654*
**Any same-sex attraction**	**6.5%**	**[5.8%-7.2%]**	**11.5%**	**[10.7%-12.2%]**
*Of those (denominators (unweighted*, *weighted))*	*468*	*486*	*1140*	*876*
**Sexual Identity**				
Heterosexual/Straight	62.4%	[57.1%-67.4%]	78.4%	[75.7%-80.9%]
Gay/Lesbian	23.0%	[18.9%-27.8%]	8.5%	[6.8%-10.5%]
Bisexual	13.0%	[9.8%-17.0%]	11.4%	[9.5%-13.6%]
Other	1.6%	[0.7%-3.6%]	1.7%	[1.0%-3.0%]
**Same-sex experience but never with genital contact**	**2.4%**	**[1.9%-2.9%]**	**5.3%**	**[4.8%-5.8%]**
*Of those (denominators (unweighted*, *weighted))*	*152*	*177*	*568*	*403*
**Sexual Identity**				
Heterosexual/Straight	92.1%	[80.1%-97.1%]	95.2%	[93.0%-96.7%]
Gay/Lesbian	0.6%	[0.1%-2.8%]	0.3%	[0.1%-1.2%]
Bisexual	6.6%	[2.0%-19.7%]	3.8%	[2.5%-5.6%]
Other	0.7%	[0.2%-2.9%]	0.8%	[0.2%-2.9%]
**Same-sex sex ever**^**a**^	**5.5%**	**[4.9%-6.2%]**	**6.1%**	**[5.6%-6.7%]**
*Of those (denominators (unweighted*, *weighted))*	*360*	*412*	*615*	*465*
**Sexual Identity**				
Heterosexual/Straight	59.5%	[53.2%-65.4%]	65.3%	[60.6%-69.7%]
Gay/Lesbian	26.3%	[21.4%-31.9%]	15.9%	[12.8%-19.6%]
Bisexual	13.8%	[10.1%-18.5%]	17.1%	[13.9%-20.9%]
Other	0.4%	[0.1%-1.3%]	1.7%	[0.8%-3.5%]
**Opposite-sex sex ever**^**b**^	**96.1%**	**[95.5%-96.5%]**	**97.0%**	**[96.7%-97.4%]**
*Of those (denominators (unweighted*, *weighted))*	*5911*	*7199*	*8501*	*7425*
**Sexual Identity**				
Heterosexual/Straight	98.1%	[97.6%-98.4%]	97.4%	[97.1%-97.8%]
Gay/Lesbian	0.7%	[0.6%-1.0%]	0.9%	[0.7%-1.1%]
Bisexual	1.0%	[0.7%-1.4%]	1.4%	[1.2%-1.7%]
Other	0.2%	[0.1%-0.4%]	0.3%	[0.2%-0.5%]
**Same- and opposite-sex sex ever**^**c**^	**4.7%**	**[4.1%-5.3%]**	**5.9%**	**[5.4%-6.5%]**
*Of those (denominators (unweighted*, *weighted))*	*296*	*350*	*593*	*451*
**Sexual Identity**				
Heterosexual/Straight	69.7%	[63.2%-75.5%]	67.2%	[62.4%-71.6%]
Gay/Lesbian	14.6%	[10.9%-19.3%]	13.7%	[10.8%-17.3%]
Bisexual	15.3%	[11.1%-20.8%]	17.4%	[14.0%-21.3%]
Other	0.4%	[0.1%-1.5%]	1.7%	[0.8%-3.6%]

^a^ Same-sex experience with genital contact

^b^ Opposite-sex experience with genital contact

^c^ Same- and opposite-sex experience with genital contact

### Dimensions of sexual orientation by recency of same-sex sex

Overall, 2·6% of men and 3·2% of women aged 16–74 years reported same-sex sex in the past five years (S2 Table). Approximately 2·9% of men and women (16–74 years) reported same-sex sex ever, but not in the past 5 years. Less than half of men and around 70% of women who reported same-sex sex ever, but not in the past 5 years, reported any same-sex attraction, compared to more than 80% of those who reported same-sex sex in the past 5 years. More than 85% of those who reported same-sex sex ever, but not in the past 5 years, reported opposite-sex sex in the past 5 years. However, opposite-sex sex was also common among those who reported recent same-sex sex; 44·0% of men and 72·1% of women reporting same-sex sex in the past 5 years also reported opposite-sex sex in that period. Younger women who reported same-sex sex in the past 5 years were more likely to self-identify as bisexual and less likely to identify as lesbian/gay than older women: they were also more likely to report opposite-sex sex in the past 5 years.

### Overlap of dimensions of sexual orientation

**Fig 2:**
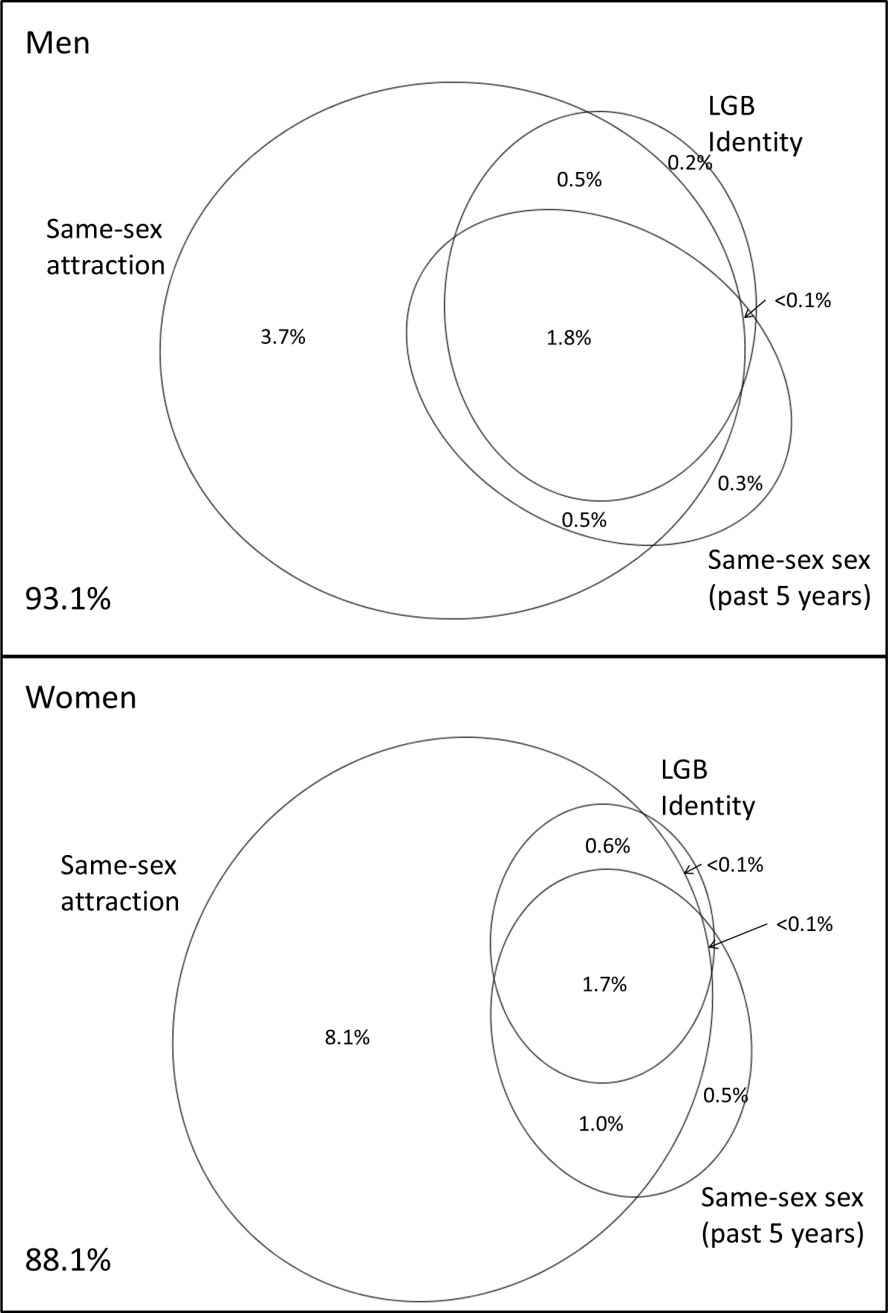
The overlap between same-sex sexual attraction (ever), LGB sexual identity and same-sex sex within the past 5 years for men and women.

Substantial incongruity exists between the three measures of sexual orientation at an individual level (Fig 2) [13], particularly for women. Around 1·7% of men and women self-identified as LGB and reported same-sex attraction and had had same-sex sex in the past 5 years. However, a higher percentage of women than men reported same-sex sex and/or same-sex attraction without reporting an LGB identity.

### Estimates of the size of sexual minority populations in 16–74 year olds in Britain in 2011

The size of the population in Britain who report lesbian, gay or bisexual identity is substantially smaller than the population reporting any same-sex attraction or sex (Table 3). When the survey was completed in 2011, there would have been an estimated 547,000 men who identified as gay or bisexual and this increases by 657,000 to 1,204,000 reporting ever having had same-sex sex and then to 1,423,000 for those reporting same-sex attraction. Similarly, 546,000 women are estimated to have identified as lesbian or bisexual and this increases by 843,000 to 1,389,000 ever having had same-sex sex and then to 2,618,000 for those reporting same-sex attraction. Estimates of the size of the male population in Britain reporting same-sex sex in the past 5 years are similar to those reporting gay or bisexual. However, for women, the former population is larger.

**Table 3:** Estimates of size of sexual minority populations in Britain for men and women aged 16–74 years.

	Men		Women	
	Natsal-3 estimate of prevalence	Estimated no. people^a^	Natsal-3 estimate of prevalence	Estimated no. people^a^
Number of people aged 16–74 years in Britain from Census data		**21,896,653**		**22,765,718**
**Sexual identity**				
Heterosexual/Straight	97.1%	21,261,650	97.3%	22,151,044
Gay/Lesbian	1.5%	328,450	1.0%	227,657
Bisexual	1.0%	218,967	1.4%	318,720
*Total LGB*	*2*.*5%*	*547*,*416*	*2*.*4%*	*546*,*377*
Other sexual identities	0.3%	65,690	0.3%	68,297
**Sexual Attraction**				
Opposite sex only	93.0%	20,363,887	87.8%	19,988,300
More often opposite sex, at least once same sex	4.0%	875,866	9.2%	2,094,446
About equal	0.6%	131,380	1.1%	250,423
More often same sex, at least once opposite sex	1.0%	218,967	0.8%	182,126
Same sex only	0.9%	197,070	0.4%	91,063
*Total any same-sex attraction*	*6*.*5%*	*1*,*423*,*282*	*11*.*5%*	*2*,*618*,*058*
Never felt sexually attracted to anyone	0.5%	109,483	0.8%	182,126
**Sexual Experience**				
Opposite sex only	91.7%	20,079,231	88.0%	20,033,832
More often opposite sex, at least once same sex	4.8%	1,051,039	9.3%	2,117,212
About equal	0.3%	65,690	0.6%	136,594
More often same sex, at least once opposite sex	1.0%	218,967	0.6%	136,594
Same sex only	0.7%	153,277	0.2%	45,531
*Total any same-sex experience*	*6*.*8%*	*1*,*488*,*972*	*10*.*7%*	*2*,*435*,*932*
Never had sexual experience with anyone	1.5%	328,450	1.3%	295,954
**Same-sex experience without genital contact**	2.4%	525,520	5.3%	1,206,583
**Same-sex sex ever**^**b**^	5.5%	1,204,316	6.1%	1,388,709
**Recent same-sex sex (past 5 years)**	2.6%	569,313	3.2%	728,503

^a^Estimates obtained by multiplying ONS 2011 census population estimates of number of people aged 16–74 years in British population by prevalence estimates from Natsal-3

^b^Same-sex experience with genital contact ever

## Discussion

Data from a national probability sample survey have shown that estimates of the size of sexual minority populations depend on the dimension of sexual orientation used, with more people reporting same-sex attraction (6·5% of men and 11·5% of women) or behaviour (5·5% of men and 6·1% of women reporting same-sex sex ever and 2·6% of men and 3·2% of women reporting same-sex sex in the past 5 years) than LGB identity (2·5% of men and 2·4% of women). Larger population estimates are therefore obtained when applying the dimension of same-sex attraction or same-sex sex ever, while smaller numbers correspond to LGB identities and more recent same-sex sex.

Natsal-3 is unique within Britain in collecting representative data on sexual identity, attraction and behaviour. As in other studies, we observed that the different dimensions of sexual orientation identify different groups of people[14,15]. Organisations that provide data on, or services for, different population groups should therefore consider whether their purposes are best served by using definitions based on sexual identity or same-sex attraction and/or behaviour. We summarise the strengths and weaknesses of each measure in Box 2 to assist decision-making regarding the relative merits of each measure. More than a quarter of men and approximately half of women reporting same-sex sex in the past 5 years identified as heterosexual, which is why a focus on behaviour rather than identity or attraction is preferable in epidemiological studies of the transmission of sexually transmitted infections (STI) and HIV and for those concerned with sexual risk behaviours[16,17]. For example, sexual health services targeting gay men may be missed by men who have had recent same-sex sex but who identify as heterosexual or bisexual. Alternatively, organisations challenging discrimination and promoting rights may find sexual identity a more pertinent categorisation.

**Box 2. Characteristics of different measures of sexual orientation**

Sexual Identity:

-     Measure felt to be most closely related to discrimination and stigma
-     Easiest to collect data on
-     Will miss many people engaging in same-sex behaviour or reporting same-sex attraction

Same-sex behaviours:

-     Useful for e.g. sexual health services where behaviour is predictive of risk; modelling STI/HIV interventions
-     Will depend on behaviours and time-frame considered
-     More recent same-sex behaviour will map more closely onto current sexual identity
-     Questions on sexual behavior may be perceived to be more sensitive any may therefore not always be appropriate

Same-sex sexual attraction:

-     Will provide largest estimate of population size
-     Would miss only a small number of people identifying as gay, lesbian or bisexual or engaging in same-sex behaviour
-     Will include many people not reporting same-sex sex and/or LGB identity
-     May not be very specific for targeting purposes

The ONS suggests that sexual identity is the component of sexual orientation most closely related to experiences of disadvantage and discrimination, and that collecting data on this aspect of sexual orientation will enable organisations to meet equality legislation[1]. It is perhaps for these reasons that a number of national surveys in Britain collect data on sexual identity using wording which is identical or very similar to the ONS question[18]. A recent report combined the results of data from 15 surveys, which had used this question and estimated that 2·5% (weighted mean) of the English population self-identified as LGB or ‘other’, compared to 2·8% in Natsal-3 [19]. However, estimates varied substantially between surveys. It is important to consider the practical aspects of asking these questions, especially in terms of social acceptability. Studies whose primary purpose is unrelated to sexual attitudes report higher non-response for this question (1·3%-14·7% vs. 0·3% in Natsal-3)[18] and many estimate a lower prevalence of LGB identities; in 2009–2010, 1·6% of participants in the Integrated Household Survey described their sexual identity as lesbian, gay or bisexual, compared to 2·5% in Natsal-3 (2010–2012)[18]. Lower non-response and higher reporting of sensitive sexual behaviours was also seen in Natsal-3 in a comparison of responses to sexual behaviour questions included in the Health Survey for England (HSE), a nationally-representative general health survey[20]. Thus differences in the primary purpose of the surveys and therefore the context in which these sensitive questions are being asked, are likely partly responsible for variation in estimates. However, differences in sample composition and survey methodologies may also be responsible for variation, as we have demonstrated by comparing estimates from Natsal and web panels[21].

As in the ONS question, sexual identity is often categorised as heterosexual/straight, gay/lesbian, bisexual or other. However, while these categories are widely used, sexual orientation does not always fit into such definable categories and may be better expressed on a continuum[22–24]. Furthermore, there is evidence that sexual orientation, or dimensions of it, is relatively labile and this may be especially true for women[25–27]. The wording of the ONS question on sexual identity (“Which of the options [on this card] best describes how you think of yourself?”) used in the Natsal-3 survey, allows for this, aiming to capture current, rather than fixed, sexual identity by using the present tense. Longitudinal study designs are better able to capture fluidity in reporting of sexual identity over time[28] but this is not possible at an individual level in a cross-sectional survey like Natsal-3, which captures participants’ opinions and experience at the time of interview. However, the questions on sexual attraction and experience asked in Natsal-3 go some way towards portraying the spectrum of attraction and behaviour experienced by participants to date (rather than specifically at the time of the interview) as both were worded in the past tense. Less than half of those who reported same-sex sex more than five years ago reported any same-sex attraction, compared to more than 80% of those who reported same-sex sex in the past 5 years. This may reflect participants’ interpretation of temporality in the question, or may reflect recall bias (that it may be easier to recall an experience than a feeling) or changes in interpretation of past experiences and feelings or a greater need to feel congruent between behaviour and attraction with more recent behaviour. Same-sex experience may be regarded as a highly-sensitive behaviour, making people unwilling to respond to questions about it, even in a sexual health survey like Natsal. Including the sexual attraction and experience scales in the CASI section of the Natsal-3 questionnaire may have led to higher levels of reporting of same-sex attraction and experience since higher reporting of sexual behaviours has been seen with CASI[29]. However, only those reporting sexual experience were eligible to complete the CASI section, and therefore situating these scales in the CAPI section allowed us to calculate estimates of attraction and identity unrestricted by sexual activity. Minority groups are represented in small numbers, even in large surveys, which limits the extent to which survey data can be used to make inter-group comparisons. However, valid descriptive statistics can still be calculated.

In common with other research[30–33], we demonstrate higher prevalences of LGB identities, same-sex attraction and same-sex sex in younger than older women and an increase in same-sex sex for women over time[10]. These changes were not seen for men. Research suggests that female sexuality is more fluid than male sexuality: both over the lifecourse, with experience of same-sex behaviour particularly likely when young[34]; and, particularly in the face of societal shifts[35]. Societal attitudes may be more permissive to same-sex sexuality between women than men[36,37]. Furthermore, reporting of same-sex and opposite-sex attractions tend to be more mutually exclusive in men than women[38] which may explain the greater degree of overlap in the dimensions of sexual orientation for men than women.

There are documented health disparities, particularly in sexual and mental health, between those identifying as LGB and those identifying as heterosexual[3,4,6]. There is also an increasing demand for data on sexual orientation to meet equality legislation. Data on the size of populations with different sexual orientations and their health outcomes will facilitate understanding of the health and wellbeing needs of particular groups, monitoring of potential inequities and planning and commissioning of services. The most appropriate choice of dimension depends on context and purpose and the decision to use a particular dimension should be made explicitly, with a clear rationale, and with awareness of the limitations of each.

## Supporting information

S1 Table Sexual attraction and experience among men and women aged 16–74 years, Britain, 2010–12. (DOCX)

S2 Table Sexual identity, same-sex attraction and recent opposite-sex sex among men and women reporting same-sex sex ever, by recency of same-sex sex and age, Britain, 2010–12. (DOCX)
