# Supplementary material for: Sexual identity, attraction and behaviour in Britain: The implications of using different dimensions of sexual orientation to estimate the size of sexual minority populations and inform public health interventions
Source: PLoS One. 2018 Jan 2;13(1):e0189607. doi: 10.1371/journal.pone.0189607 (PMC5749676; doi:10.1371/journal.pone.0189607)
Supplement: S1 Table — (DOCX) [file pone.0189607.s001.docx]

| **S1 Table: Sexual Attraction and Experience Among Men and Women Aged 16-74 years, Britain, 2010-12** | | | | | | | | |  |  |  |  |
| --- | --- | --- | --- | --- | --- | --- | --- | --- | --- | --- | --- | --- |
|  |  |  |  |  |  |  |  |  |  |  |  |  |
|  | **Men** | | | | | | **Women** | | | | | |
|  | 16-34 | | 35-74 | | Total | | 16-34 | | 35-74 | | Total | |
| *Denominators (unweighted, weighted)* | *3246* | *2606* | *3017* | *4867* | *6293* | *7474* | *4613* | *2578* | *4230* | *5049* | *8843* | *7628* |
|  | % | 95%CI | % | 95%CI | *%* | *95%CI* | % | 95%CI | % | 95%CI | *%* | *95%CI* |
| **Sexual attraction** |  |  |  |  |  |  |  |  |  |  |  |  |
| Opposite sex only | 91.8% | 90.7, 92.7 | 93.7% | 92.8, 94.6 | *93.0%* | *92.3, 93.7* | 81.7% | 80.4, 82.9 | 90.9% | 89.9, 91.8 | *87.8%* | *87.0, 88.5* |
| More often opposite sex, and at least once same sex | 4.3% | 3.7, 5.1 | 3.8% | 3.2, 4.6 | *4.0%* | *3.5, 4.6* | 13.9% | 12.8, 15.1 | 6.9% | 6.1, 7.7 | *9.2%* | *8.6, 9.9* |
| About equally often to opposite sex and same sex | 0.6% | 0.4, 1.0 | 0.6% | 0.4, 0.9 | *0.6%* | *0.4, 0.8* | 2.0% | 1.6, 2.5 | 0.7% | 0.4, 1.0 | *1.1%* | *0.9, 1.4* |
| More often same sex, and at least once opposite sex | 1.2% | 0.9, 1.8 | 0.9% | 0.6, 1.3 | *1.0%* | *0.8, 1.3* | 0.9% | 0.7, 1.3 | 0.7% | 0.5, 1.0 | *0.8%* | *0.6, 1.0* |
| Same sex only | 1.2% | 0.9, 1.7 | 0.7% | 0.4, 1.1 | *0.9%* | *0.7, 1.2* | 0.4% | 0.2, 0.6 | 0.4% | 0.2, 0.6 | *0.4%* | *0.3, 0.6* |
| Never felt sexually attracted to anyone | 0.8% | 0.5, 1.3 | 0.3% | 0.2, 0.5 | *0.5%* | *0.3, 0.7* | 1.2% | 0.8, 1.7 | 0.6% | 0.4, 0.8 | *0.8%* | *0.6, 1.0* |
|  |  |  |  |  |  |  |  |  |  |  |  |  |
| **Sexual experience** |  |  |  |  |  |  |  |  |  |  |  |  |
| Opposite sex only | 89.4% | 88.2, 90.6 | 93.0% | 91.9, 93.9 | *91.7%* | *90.9, 92.5* | 78.6% | 77.1, 80.0 | 92.9% | 92.0, 93.6 | *88.0%* | *87.3, 88.8* |
| More often opposite sex, and at least once same sex | 4.9% | 4.1, 5.8 | 4.8% | 4.0, 5.7 | *4.8%* | *4.2, 5.5* | 16.0% | 14.8, 17.3 | 5.8% | 5.1, 6.6 | *9.3%* | *8.6, 10.0* |
| About equally often to opposite sex and same sex | 0.4% | 0.2, 0.6 | 0.2% | 0.1, 0.5 | *0.3%* | *0.2, 0.4* | 1.0% | 0.7, 1.3 | 0.3% | 0.2, 0.6 | *0.6%* | *0.4, 0.8* |
| More often same sex, and at least once opposite sex | 1.2% | 0.9, 1.7 | 0.9% | 0.6, 1.3 | *1.0%* | *0.8, 1.3* | 0.7% | 0.5, 1.1 | 0.6% | 0.4, 0.9 | *0.6%* | *0.5, 0.8* |
| Same sex only | 0.9% | 0.6, 1.4 | 0.6% | 0.4, 0.9 | *0.7%* | *0.5, 1.0* | 0.2% | 0.1, 0.3 | 0.2% | 0.1, 0.4 | *0.2%* | *0.1, 0.3* |
| Never had sexual experience with anyone | 3.2% | 2.6, 3.9 | 0.5% | 0.3, 0.8 | *1.5%* | *1.2, 1.8* | 3.5% | 2.8, 4.3 | 0.2% | 0.1, 0.4 | *1.3%* | *1.1, 1.6* |
| **P*<0.001 comparing age groups for women for both sexual attraction and sexual experience | | | | | | | | | | | | |
