# Supplementary material for: Sexual identity, attraction and behaviour in Britain: The implications of using different dimensions of sexual orientation to estimate the size of sexual minority populations and inform public health interventions
Source: PLoS One. 2018 Jan 2;13(1):e0189607. doi: 10.1371/journal.pone.0189607 (PMC5749676; doi:10.1371/journal.pone.0189607)
Supplement: S2 Table — (DOCX) [file pone.0189607.s002.docx]

| **S2 Table: Sexual Identity, Same-sex Attraction and Recent opposite-sex Sex Among Men and Women Reporting Same-sex Sex Ever, by Recency of Same-sex Sex and Age, Britain, 2010-12** | | | | | | | | | | | | |
| --- | --- | --- | --- | --- | --- | --- | --- | --- | --- | --- | --- | --- |
|  | **Men** | | | | | | **Women** | | | | | |
|  | 16-34 | | 35-74 | | Total | | 16-34 | | 35-74 | | Total | |
| *Denominators (unweighted, weighted)* | *3254* | *2613* | *3039* | *4895* | *6293* | *7508* | *4627* | *2587* | *4242* | *5067* | *8869* | *7654* |
| **Same-sex sex in the past 5 years** | **3.2%** | **2.6, 3.9** | **2.2%** | **1.7, 2.9** | ***2.6%*** | ***2.1, 3.0*** | **5.4%** | **4.7, 6.2** | **2.0%** | **1.7, 2.5** | ***3.2%*** | ***2.8, 3.6*** |
| *Of those (denominators (unweighted, weighted)):* | *117* | *83* | *73* | *108* | *190* | *191* | *260* | *139* | *91* | *103* | *351* | *242* |
| **Sexual Identity** |  |  |  |  |  |  |  |  |  |  |  |  |
| Heterosexual/Straight | 21.3% | 13.6, 31.7 | 34.1% | 22.5, 47.9 | *28.4%* | *20.8, 37.5* | 49.9% | 42.9, 56.9 | 38.1% | 27.8, 49.6 | *44.8%* | *38.7, 51.1* |
| Gay/Lesbian | 61.2% | 50.4, 71.0 | 45.7% | 33.1, 58.8 | *52.5%* | *43.6, 61.2* | 18.2% | 13.4, 24.3 | 42.5% | 32.3, 53.3 | *28.6%* | *23.2, 34.6* |
| Bisexual | 16.5% | 10.2, 25.6 | 20.3% | 12.0, 32.2 | *18.6%* | *12.8, 26.3* | 31.3% | 24.9, 38.5 | 16.4% | 9.8, 26.2 | *24.9%* | *20.0, 30.6* |
| Other | 1.0% | 0.3, 4.1 | 0.0% |  | *0.5%* | *0.1, 1.8* | 0.6% | 0.1, 2.7 | 3.1% | 1.0, 9.4 | *1.7%* | *0.6, 4.3* |
|  |  |  |  |  |  |  |  |  |  |  |  |  |
| Any same-sex attraction | 91.5% | 82.5, 96.1 | 85.7% | 72.9, 93.0 | *88.3%* | *80.4, 93.2* | 87.5% | 82.8, 91.0 | 80.2% | 68.7, 88.2 | *84.3%* | *78.8, 88.6* |
|  |  |  |  |  |  |  |  |  |  |  |  |  |
| Had opposite-sex sex in the past 5 years | 43.5% | 33.7, 53.9 | 44.3% | 32.1, 57.3 | *44.0%* | *35.4, 52.9* | 85.3% | 79.8, 89.6 | 53.8% | 42.5, 64.7 | *72.1%* | *65.9, 77.5* |
|  |  |  |  |  |  |  |  |  |  |  |  |  |
| **Same-sex sex ever but not in the past 5 years** | **1.7%** | **1.2, 2.3** | **3.6%** | **3.0, 4.4** | ***2.9%*** | ***2.5, 3.5*** | **2.8%** | **2.3, 3.5** | **2.9%** | **2.4, 3.6** | ***2.9%*** | ***2.5, 3.4*** |
| *Of those (denominators (unweighted, weighted)):* | *49* | *44* | *120* | *176* | *169* | *219* | *135* | *74* | *127* | *148* | *262* | *222* |
| **Sexual Identity** |  |  |  |  |  |  |  |  |  |  |  |  |
| Heterosexual/Straight | 91.1% | 78.5, 96.6 | 85.4% | 77.5, 90.8 | *86.5%* | *79.9, 91.2* | 90.2% | 82.9, 94.6 | 86.6% | 78.8, 91.8 | *87.8%* | *81.8, 92.0* |
| Gay/Lesbian | 0.0% |  | 4.3% | 2.0, 8.8 | *3.4%* | *1.6, 7.1* | 0.0% |  | 3.2% | 1.3, 7.7 | *2.2%* | *0.9, 5.2* |
| Bisexual | 7.2% | 2.4, 19.9 | 10.3% | 5.7, 17.9 | *9.7%* | *5.7, 15.9* | 9.1% | 4.9, 16.5 | 8.0% | 4.1, 14.9 | *8.4%* | *5.0, 13.7* |
| Other | 1.8% | 0.2, 11.6 | 0.0% |  | *0.4%* | *0.0, 2.5* | 0.6% | 0.1, 4.3 | 2.2% | 0.6, 8.0 | *1.7%* | *0.5, 5.4* |
|  |  |  |  |  |  |  |  |  |  |  |  |  |
| Any same-sex attraction | 38.3% | 24.1, 54.9 | 43.0% | 33.3, 53.2 | *42.0%* | *33.6, 51.0* | 69.2% | 59.5, 77.5 | 67.7% | 58.0, 76.1 | *68.2%* | *61.0, 74.7* |
|  |  |  |  |  |  |  |  |  |  |  |  |  |
| Had opposite-sex sex in the past 5 years | 91.6% | 79.3, 96.9 | 85.0% | 78.1, 90.1 | 86.3% | 80.6, 90.6 | 99.1% | 96.2, 99.8 | 86.5% | 79.3, 91.4 | *90.7%* | *85.9, 94.0* |
| p-value comparing those who've had same-sex sex ever but not in the past 5 years to those who have had same-sex sex in the past 5 years were <0.001 for sexual identity, any same-sex attraction and had opposite-sex sex in the past 5 years for men and women for all ages with the exception of any same-sex attraction for women aged 35-74 years. | | | | | | | | | | | | |
| p-values for a difference by age were <0.001 for women reporting same-sex sex in the past 5 years for sexual identity and had opposite-sex sex in the past 5 years; and for women reporting same-sex sex but not in the past 5 years for had opposite-sex sex in the past 5 years | | | | | | | | | | | | |
